# In vitro evaluation of a ceramic bracket with a laser-structured base

**DOI:** 10.1186/s12903-020-1009-9

**Published:** 2020-01-21

**Authors:** Selma Elekdag-Türk

**Affiliations:** Faculty of Dentistry, Department of Orthodontics, University of Ondokuz Mayis, 55139 Kurupelit, Samsun, Turkey

**Keywords:** Ceramic bracket, Laser-structured base, Shear bond strength

## Abstract

**Background:**

The purpose of this study was the assessment of shear bond strength (SBS), adhesive remnant characteristics, integrity of the enamel, integrity of Discovery Pearl as well as the integrity of Fascination 2 ceramic brackets following SBS testing.

**Methods:**

Sixty maxillary first premolars were randomly assigned into two groups. These groups were bonded with their respective brackets. The samples underwent thermocycling (1000 cycles), SBS testing and assessment of the residual adhesive. The statistical analyses used were the independent samples t-test, the Weibull analysis and the chi-square test.

**Results:**

The independent samples t-test for the comparison of the mean SBS resulted in significant differences between Fascination 2 (10.50 ± 2.61 MPa) and Pearl (13.01 ± 2.50 MPa) brackets (*p* = 0.0003). The results of the chi-square test for ARI demonstrated a significant difference (*p* = 0.000) between the groups. A higher frequency of ARI scores of 2 and 3 for Pearl brackets existed. Enamel damage and bracket fracturing was not observed.

**Conclusions:**

The mean bond strength value, the adhesive remnant characteristics, the integrity of the enamel and the ceramic brackets as well as the Weibull analyses outcomes were highly encouraging during this in vitro screening. The way is paved for an in vivo investigation with the Pearl ceramic bracket.

## Background

Esthetic brackets, composed of monocrystalline or polycrystalline ceramic materials, were introduced approximately 30 years ago [1–3]. These tooth-colored, more socially acceptable, magnetic resonance imaging safe brackets [1, 4] with excellent biocompatibility [1, 5] have gained widespread popularity since their introduction [1–3].

Ceramic materials are inert and do not bond chemically with adhesives. Therefore, ceramic brackets may acquire their bond strength (BS) from three different types of retention mechanisms: a chemical retention mechanism (by means of a silane coupling agent), a mechanical retention mechanism or a combination of both retention mechanisms [1, 6].

For the chemical retention method, glass is attached to the smooth ceramic bracket base which is subsequently treated with a silane coupler. This silane molecule is a bifunctional molecule, that is, one end of this molecule bonds with the flat glass layer on the bracket base, while the other end of this molecule reacts with the orthodontic adhesive [1, 6]. However, for the mechanical retention mechanism retentive indentations or undercuts are created on the bracket base. These retentive indentations or undercuts provide a mechanical interlocking with the orthodontic adhesive [1, 6].

It was pointed out that most ceramic bracket manufacturers have shifted away from the chemical retention mechanism, since purely chemical retention has been stated to yield a remarkably high BS that might harm the enamel surface during the debracketing procedure. In fact, purely chemical retention has become obsolete [1, 2]. Nowadays, the majority of ceramic brackets solely rely on mechanical retention or on a combination of both retention mechanisms [1, 2].

The Fascination® 2 (Dentaurum, Ispringen, Germany) ceramic bracket was introduced more than a decade ago. This type of bracket incorporates a silane coated button-structured base. Thereby, relying on a combination of both retention mechanisms, i.e. chemical as well as mechanical [2]. Theoretically, the incorporation of these ‘knobs’ was to ensure a thicker layer of adhesive when compared to the flat base design of the chemically retained ceramic precursors. It had been reported that a thicker layer of adhesive reduces shear bond strength (SBS) [7] and thereby lowering the risk of enamel damage. An in vitro study comparing Fascination® 2 with flat, silane treated bases (purely chemical retention) demonstrated that Fascination® 2 brackets provided significantly lower, yet clinically acceptable SBS values [8] The Fascination 2 bracket is currently being used internationally by orthodontic clinicians.

Nowadays a plethora of ceramic brackets with different base designs are available [1]. The Discovery® Pearl ceramic bracket (Dentaurum, Ispringen, Germany) is a recently introduced ceramic bracket with an innovative laser-structured base. The manufacturer of this bracket, solely relying on mechanical retention, claims consistent and adequate BS as well as a safe debonding process. This study will serve as a precursor to a controlled clinical investigation. To date, no in vitro study with human dental enamel has evaluated the performance of this bracket.

The objective was the assessment of the following parameters:

1. the SBS values,
2. the adhesive remnant characteristics,
3. the integrity of the enamel surfaces,
4. the integrity of Fascination 2 and Discovery Pearl ceramic brackets following machine debracketing as well as
5. the Weibull survival analysis for Fascination 2 and Discovery Pearl ceramic brackets.

The null hypothesis was that there would be no difference in the aforementioned parameters.

## Methods

Sixty human maxillary first premolars from orthodontic extraction patients, 14–16 years old, composed this study. Written as well as verbal consent was obtained from all parents and patients. These teeth stemmed from patients from an area with low fluoride concentration (≤0.05 ppm) in the public water supply. Furthermore, intact premolars (absence of caries, restorations and hypoplasia) with minimum crown contour variations, no extraction damage and no history of pre-treatment with any chemical agents were included. Following extraction, the teeth were cleaned under tap water for soft tissue and debris removal. Subsequently, they were placed into a 0.1% (%) thymol solution (weight/volume). Thymol storage period of the teeth did not exceed 6 months. This antimicrobial solution was renewed on a monthly basis.

Each tooth was embedded in a cold-cure acrylic resin (Orthocryl; Dentaurum, Ispringen, Germany) cylindrical block. The buccal surface of each tooth was aligned parallel to the base of the mold with the assistance of a jig. Thereby, keeping the buccal surface of each tooth parallel to the applied force during the shear test. Subsequently, the teeth were cleansed and polished with non-fluoridated, oil-free pumice paste and rubber prophylactic cups for 10 s. After pumicing, the 60 teeth were randomly assigned into two groups, group Fascination and group Pearl. The sample size was estimated by G*Power (version 3.1.9.2.) [9] according to a previous study [10] on the subject of SBS of ceramic brackets (80% power; 5% significance level; 2-tailed). Accordingly, a minimum sample size of 17 in each group was required to detect a significant difference between the groups. Nevertheless, the sample size was increased to 30 according to the guidelines presented in a critique on BS testing [11]. This critique recommended that if sound conclusions are to be made from BS testing 30 samples should be included into each group [11].

**Fig. 1:**
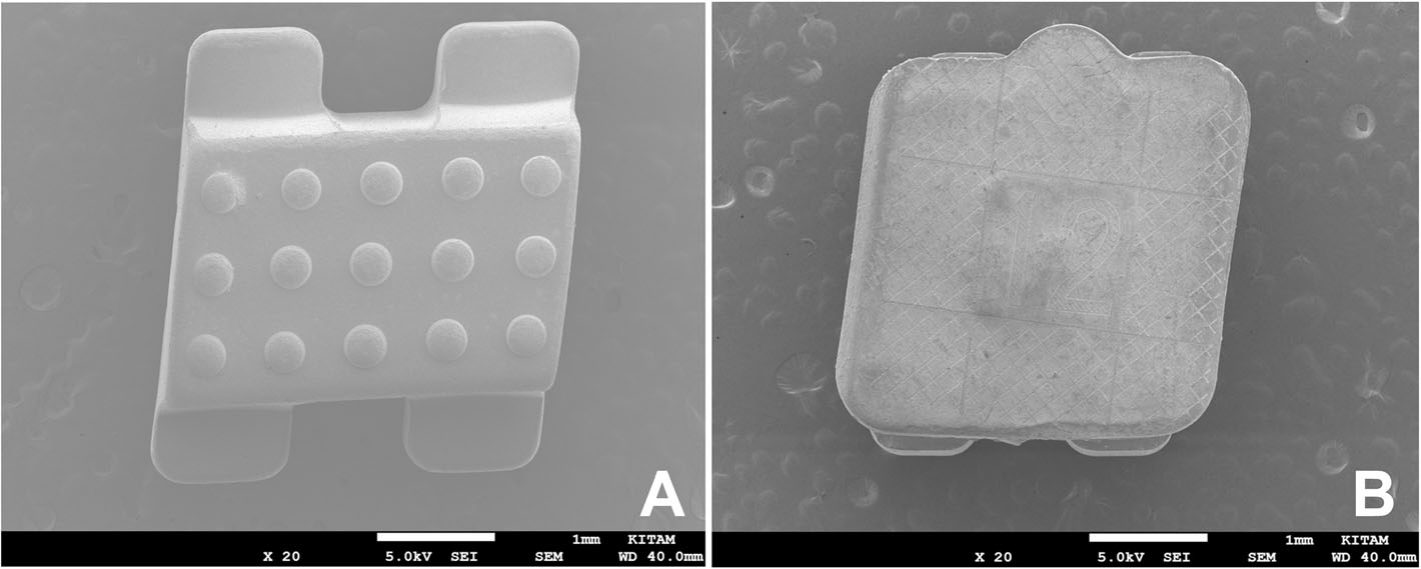
Scanning Electron Microscope images of the Fascination® 2 (**a**) and the Discovery® Pearl (**b**) bracket bases

In group Fascination (the control group), Fascination® 2 (Dentaurum, Ispringen, Germany) ceramic brackets with 0.022 in. slots were used. The bonding area according to the manufacturer was 11.02 square millimeters (mm^2^) (Fig. 1a).

In group Pearl, Discovery® Pearl (Dentaurum, Ispringen, Germany) ceramic brackets with 0.022 in. slots were used. The bonding area according to the manufacturer was 11.16 mm^2^ (Fig. 1b).

Positioning guides/aides were removed to allow for meticulous excess adhesive removal. Also, the amount of light transmitted during photocuring was not hindered [1].

For each group the bonding procedure was as follows: The teeth were etched with 37% phosphoric acid for 30 s, washed for 20 s, and dried for 10 s. After etching, a thin coat of primer (Transbond XT Primer; 3 M Unitek, Monrovia, California, USA) was applied.

The adhesive resin (Transbond XT Light Cure Adhesive Paste; 3 M Unitek) was placed onto the bracket base and each bracket was firmly seated onto the buccal surface of each tooth. Exuded excess adhesive was meticulously cleared away before curing. The adhesive resin was polymerized for 10 s from above the bracket using a visible curing unit with an output power of 600 milliWatts per square centimeter (mW/cm^2^).

Approximately 2 min after bonding, the samples were placed into distilled water at 37 degrees Celsius (°C) to prevent dehydration for 24 h. Subsequently, the samples underwent thermocycling, as the accelerated ageing test, for 1000 cycles. Thermocycling was carried out between 5 and 55 °C with a dwelling time of 30 s as advised by the International Organization for Standardization [12].

### SBS testing

**Fig. 2:**
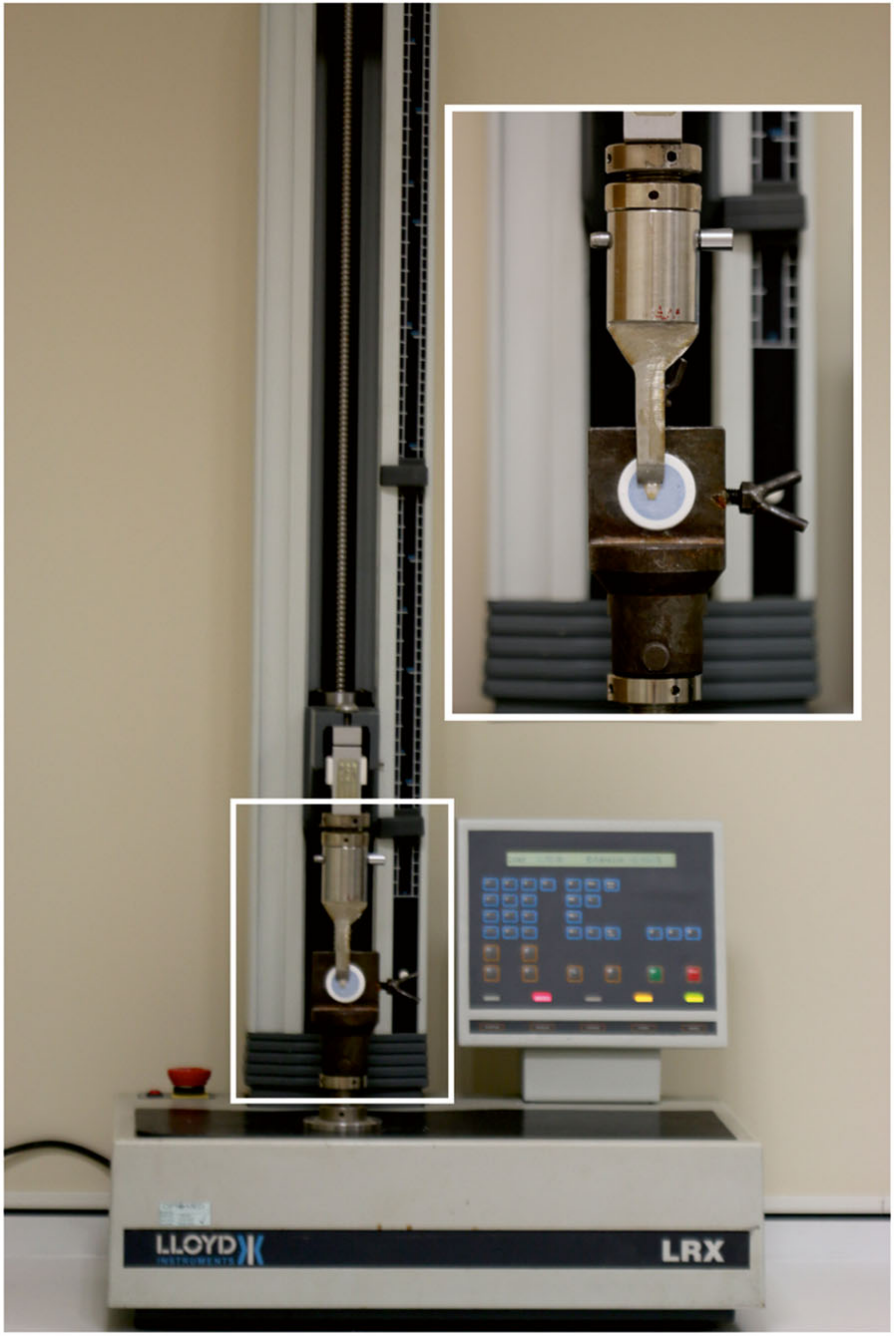
Test system for the SBS test

**Fig. 3:**
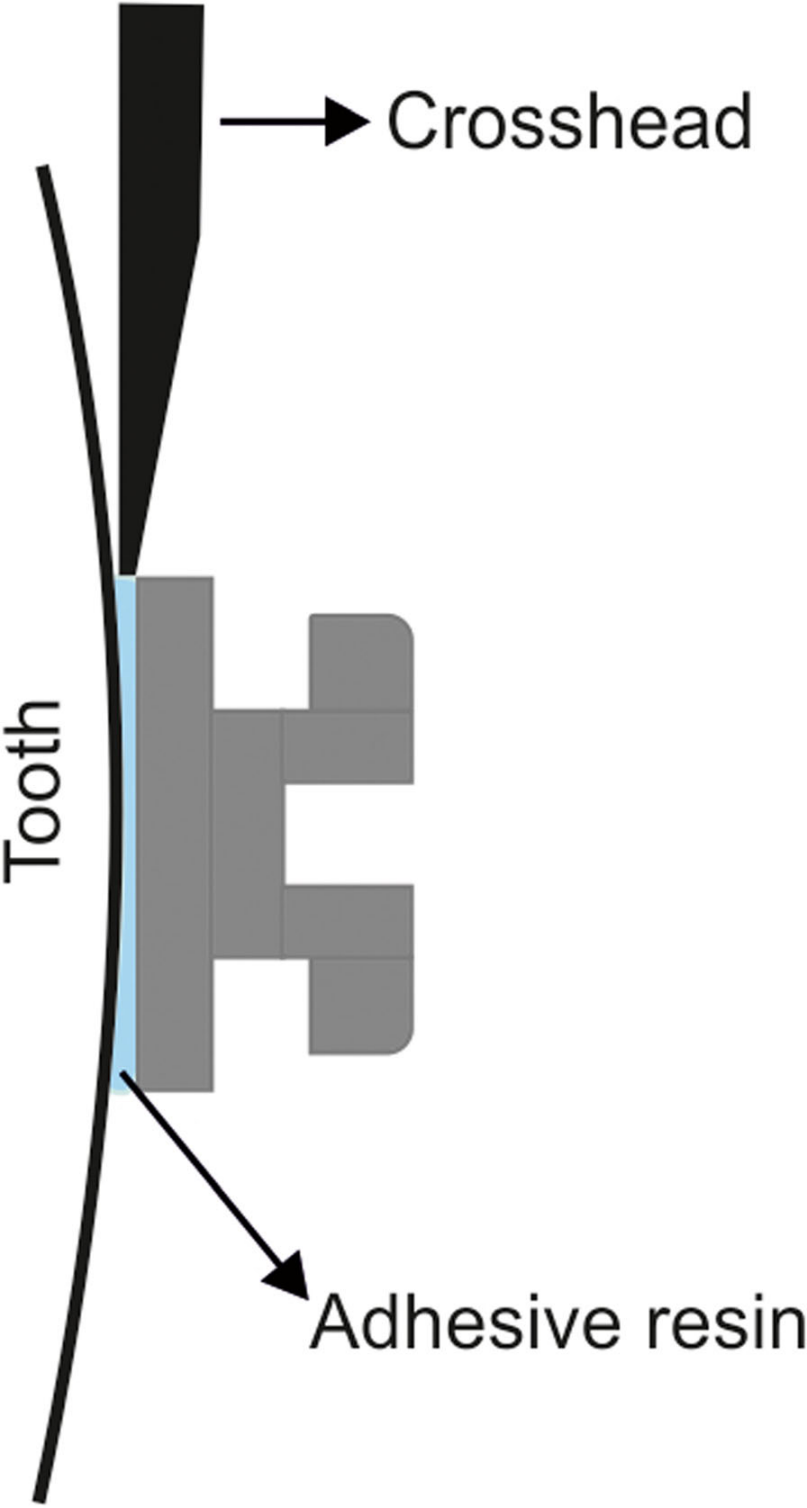
Schematic presentation of SBS testing

The SBS test was carried out with a universal testing instrument (Lloyd LRX; Lloyd Instruments Ltd., Fareham, Hants, UK). Each sample was secured in the lower part of the machine so that the shear force could be applied parallel to the bracket base. The samples were stressed in an occluso-gingival direction with a crosshead speed of 1 mm per minute (mm/min). The force was applied to the bracket base, as close to the enamel/composite interface as possible (Figs. 2 and 3). The BS, in megapascal (MPa = N/mm^2^) or newton (N) per square millimeter (mm^2^), was calculated by dividing the debonding force (N) by the bracket base surface area (mm^2^).

### Evaluation of the residual adhesive and enamel surface

Assessment of the residual adhesive and enamel surface was carried out with a stereomicroscope (Stemi 2000-C; Carl Zeiss; Göttingen, Germany) at a magnification of × 10. The amount of composite resin present on the enamel was evaluated with the adhesive remnant index (ARI). The ARI ranges from 0 to 3, with 0 signifying no adhesive remnant present on the enamel; 1, less than half of the composite left; 2, more than half of the composite left; and 3, all composite left on the enamel surface [13].

### Evaluation of brackets

The brackets were examined with a stereomicroscope (Stemi 2000-C; Carl Zeiss; Göttingen, Germany) at a magnification of × 10 to record any defects.

All procedures were carried out by the same operator. No assistance was delivered during any of the steps. Thus, inter-examiner variation was eliminated.

### Statistical analyses

The independent samples t-test was used for the comparison of the mean SBS between the two groups (*p* < 0.05).

A Weibull analysis was carried out, and the Weibull modulus, characteristic BS, correlation coefficient, and the stress levels at 5 and 10% probability of failure were determined for each group.

The chi-square test was applied to detect significant differences for the ARI scores between the groups (*p* < 0.05).

## Results

**Fig. 4:**
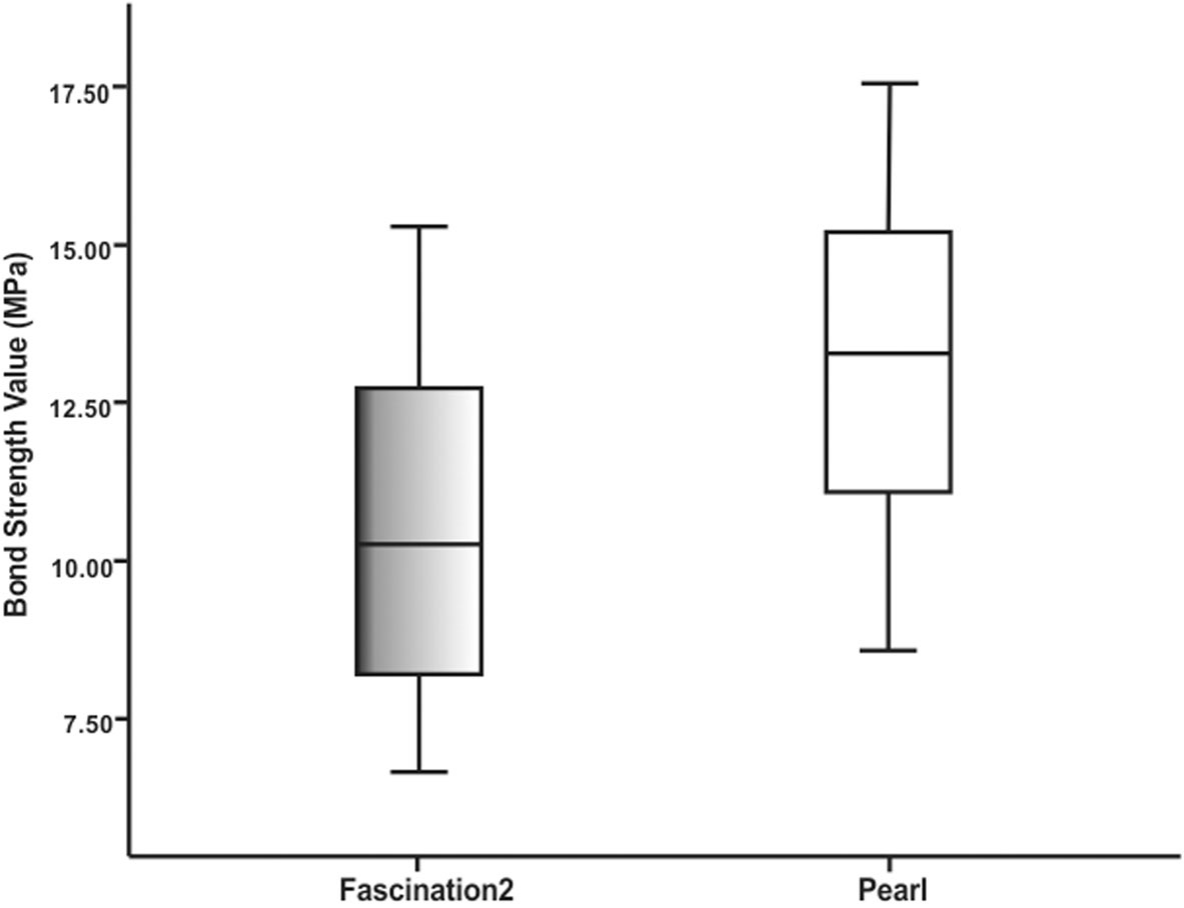
Box plot of the distribution of the bond strength values for the groups

The mean SBS, minimum and maximum values, and standard deviations for each group are presented in Table 1 and Fig. 4. The results of the independent samples t-test to compare the mean SBS are shown in Table 1. Statistically significant differences were obtained between the SBS value (10.50 ± 2.61 MPa) of Fascination 2 and the SBS value (13.01 ± 2.50 MPa) of Pearl brackets (*p* = 0.0003).

**Table 1:** Mean shear bond strengths, standard deviations (SD), minimum (Min), maximum (Max) values and Weibull parameters for each group (*n* = 30)

Groups	Mean	Min	Max	P	Weibull Analysis				
					Weibull Modulus	Correlation Coefficient	Characteristic Bond Strength (MPa)	Shear Stress at 5% Probability of Failure (MPa)	Shear Stress at 10% Probability of Failure (MPa)
Fascination 2	10.50 (2.61)	6.64	15.28	0.0003***	4.63	0.957	11.50	9.88	11.54
Pearl	13.01 (2.50)	8.58	17.54		5.93	0.980	14.02	11.38	12.85

***p = 0.001

Frequency distribution and the result of the chi-square analysis of the ARI are presented in Table 2. The outcome of the chi-squared comparisons indicated a significant difference (*p* = 0.000) for the groups. There was a higher frequency of ARI scores of 2 and 3 for the Pearl bracket.

**Table 2:** Frequency distribution and the result of the chi-square analysis of the Adhesive Remnant Index

Groups	ARI score^a^			
	0	1	2	3
Fascination 2	30	–	–	–
Pearl	3	7	10	10

*χ*^*2*^ = 49.091, *P* = 0.000

^a^Score 0 = no composite left on enamel surface; score 1 = less than half of composite left; score 2 = more than half of composite left; score 3 = all composite left on enamel surface

**Fig. 5:**
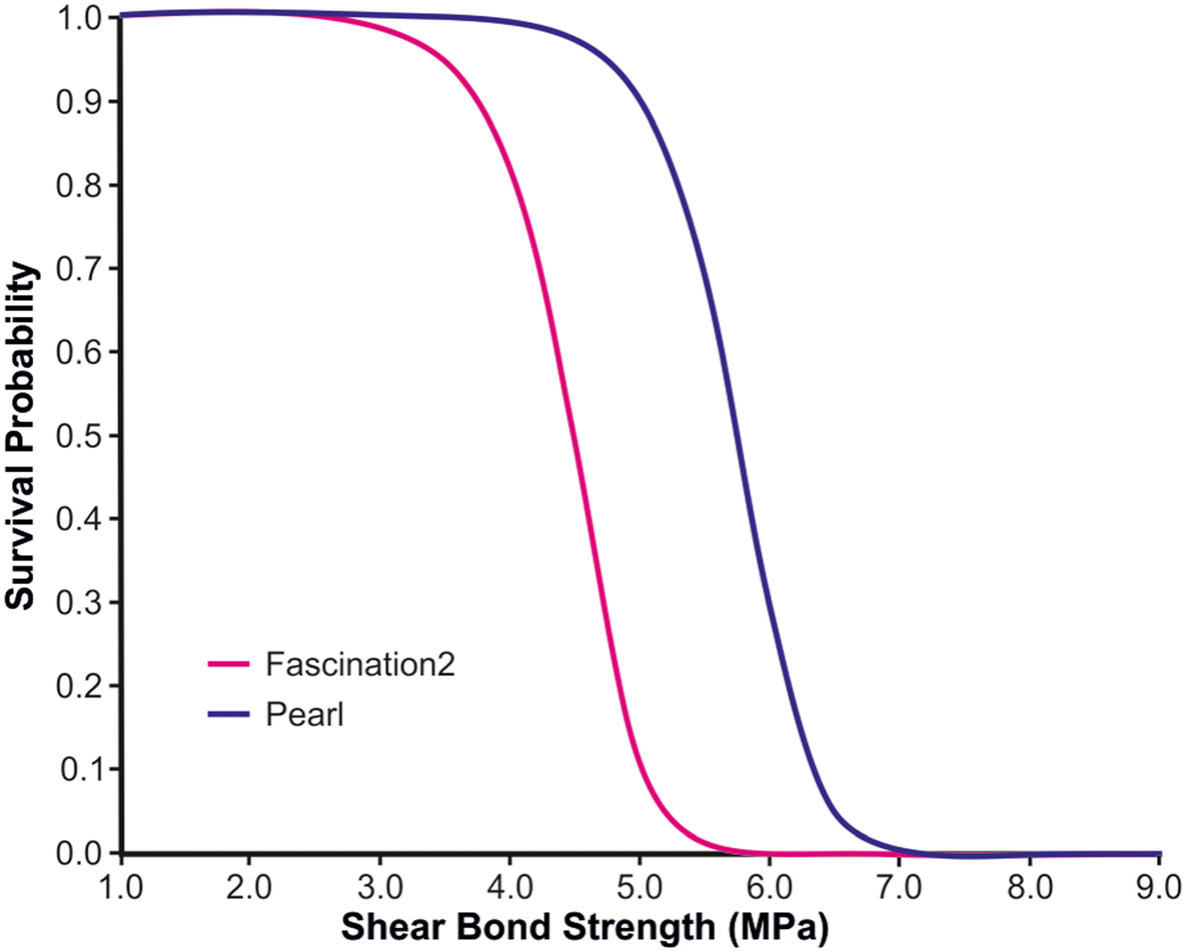
Cumulative survival probabilities versus shear bond strength for both groups

The parameters of the Weibull analysis (modulus, correlation coefficient, characteristic BS, and stress levels at 5 and 10% probability of failure) for each group are presented in Table 1. The Weibull distribution plots of the survival probability at a certain shear stress level for both groups are displayed in Fig. 5.

## Discussion

The request for more attractive orthodontic treatment has prompted orthodontic companies to develop more aesthetically pleasing appliances, such as ceramic brackets. However, in the “early” days these brackets did demonstrate a number of serious complications, such as enamel tear-outs and cracks [1–3, 14, 15].

For the past two decades, literature has reported higher BS values with chemically retained ceramic brackets when compared to mechanically retained ceramic brackets [2, 3]. Interestingly, in this study Fascination 2 ceramic brackets demonstrated a significantly lower SBS value (10.50 MPa) when compared to Discovery Pearl ceramic brackets (13.01 MPa). This outcome was completely unexpected, since Fascination 2 relies on a chemical as well as a mechanical retention mechanism, whereas Discovery Pearl relies on mechanical retention mechanism only.

The base of Fascination 2 brackets is provided with a nub-structure. The presence of these protuberances on this bracket’s base might be responsible for this unexpected outcome. It was stated that these spacers or ‘knobs’ on the Fascination 2 bracket bases were explicitly incorporated with the intent to produce a thicker layer of adhesive [8]. The adhesive industry acknowledges that thick adhesive layers produce weaker joints [16]. Furthermore, it was reported that an increase of adhesive thickness causes a decrease of SBS [7]. In fact, researchers did report a reduction in SBS of the Fascination 2 brackets when compared to its flat base precursor [8]. Thus, bracket base configuration is an important determinant concerning SBS [17]. Furthermore, the crown contour of premolar teeth, inevitably affecting the adhesive thickness, might also have contributed to the outcomes of the present study.

The quest for optimal BS means minimizing bond failures during treatment as well as minimizing the potential risks during the debonding procedure at the end of treatment. An adhesive-bracket combination should be able to resist a stress level of at least 6–8 MPa [18]. This value, described in 1975, has been considered as conjectural by some researchers [19]. On the other hand, two researchers stated that a BS of 8 MPa is excessive, and regarded 4 MPa as sufficient [20]. In the light of the present information, the mean SBS values for both types of brackets are higher than the above presented guideline values, namely 4 MPa and 8 MPa. This finding is encouraging, since researchers [21] stated that the mean in vivo BS values were approximately 40% lower than in vitro BS values. In addition, at the completion of orthodontic treatment an undamaged enamel surface following the debonding procedure should be achieved [22]. In fact, it was reported that fractures of the enamel could take place with a BS as low as 13.8 MPa [23]. Thus, 13.8 MPa is considered as the upper limit. The mean SBS values for both bracket types were within the ‘safe’ zone.

The assessment of the ARI scores presented a significant difference in bond-failure site between the two groups. Group Pearl displayed a frequency of ARI scores of 2 and 3 for two thirds of the samples. Whereas, Fascination 2 demonstrated ARI scores of 0 for all samples. The ARI scores, for the laser-structured base Pearl brackets, indicated that most of the adhesive remained on the enamel surface. This type of bond failure has the advantage of protecting the enamel surface [24, 25], yet the disadvantage of having more residual adhesive material that necessitates the mechanical removal by the orthodontist following debracketing [24]. Nevertheless, it has been pointed that the interface between the adhesive and the bracket base is the preferred and safest location of failure during the debracketing process [26].

The key point that should be emphasized is the fact that no enamel damage was observed for both groups with × 10 magnification. Theoretically, this finding was expected, since the mean SBS values were below 13.8 MPa. Yet, even with maximum values of 15.28 MPa and 17.54 MPa no enamel complications were encountered.

In addition, it has to be pointed out that teeth stored in 1% thymol solution are much drier than vital teeth and, therefore, are at a greater risk of enamel damage [27]. Furthermore, machine debracketing, when compared to manual debracketing, is extremely harsh, abrupt and unilateral. Nevertheless, unblemished enamel was observed with × 10 magnification. Furthermore, both types of brackets were intact following the harsh machine debracketing procedure, i.e., no fracturing of the ceramic brackets was observed. This finding might be anticipated for the semi-twin Fascination 2 ceramic bracket, where a ceramic connector joins the mesial and distal tie-wings. Thereby, imparting increased robustness when compared to the true-twin Pearl bracket. The ceramic injection molding production process of the Pearl bracket might also have contributed to this result [2]..

Ceramic bracket robustness as well as integrity are of utmost importance in the clinical setting. Ceramic bracket tie-wing fractures might constitute a serious problem in the oral environment, since the effective ligation of the arch wire to the impaired bracket is no longer possible. In addition, impaired brackets are prone to complete fragmentation. Ceramic fragment penetration into the oral soft tissues might ensue as well as the risk of inhalation or swallowing of these fragments by the patient does exist. Ceramic bracket fragments are not visible on radiographs. Thus, as a risk management procedure, the debonding of the impaired bracket and its replacement with a new bracket is required. This procedure is time-consuming, costly and inconvenient for the patient as well as the clinician [1, 28, 29].

The Weibull survival analysis presents data about the probability of bond failure and provides the orthodontic practitioner with important information of how the material tested, i.e. the bracket, is likely to perform in the oral environment [11]. A group of researchers [30] proposed the 5% of failure as a suitable level for BS assessment. According to these researchers [30], the BS of a material with a 5% of failure should be at least 5.4 MPa. In the current study, SBS displayed shear stress levels higher than 5.4 MPa at the 5% probability of failure for both types of brackets. This outcome implies an acceptable BS for both types of brackets under in vivo situations, i.e. the multifaceted oral environment. This outcome was anticipated for the Fascination 2 bracket. Interestingly, the Weibull plot for Discovery Pearl displayed a shift to the right. This indicates a lower probability of failure at higher levels of stress when compared to the Fascination 2 ceramic brackets.

## Conclusions

### The null hypothesis was rejected for parameters 1, 2 and 5

Unexpected bracket failure (bond failure and fracture) and particularly debracketing at the end of treatment with ceramic brackets poses an area of stress for the clinician, particularly in an ever-increasing litigious society [22]. It has been pointed out that much of the turmoil regarding ceramic brackets could have been avoided by appropriate testing procedures prior to clinical applications [31]. The BS values, the adhesive remnant characteristics, the integrity of the enamel and the ceramic brackets as well as the Weibull analyses are highly encouraging in the current in vitro screening. The way is paved for an in vivo, i.e., a clinical investigation with the Pearl ceramic bracket.

## Data Availability

All materials and data are available from the author upon request.

## Abbreviations

%: Percent
°C: Degrees Celsius
ARI: Adhesive remnant index
BS: Bond strength
mm/min: Millimeter per minute
mm^2^: Square millimeter
MPa: Megapascal
mW/cm^2^: MilliWatts per square centimeter
N/mm^2^: Newton per square millimeter
SBS: Shear bond strength
